# What’s new in pontocerebellar hypoplasia? An update on genes and subtypes

**DOI:** 10.1186/s13023-018-0826-2

**Published:** 2018-06-15

**Authors:** Tessa van Dijk, Frank Baas, Peter G. Barth, Bwee Tien Poll-The

**Affiliations:** 1 Department of Clinical Genetics, Academic Medical Center, Amsterdam, The Netherlands; 2 Department of Clinical Genetics, Leiden University Medical Center, Leiden, The Netherlands; 3 Department of Pediatric Neurology, Academic Medical Center, Amsterdam, The Netherlands

**Keywords:** Pontocerebellar hypoplasia, Pediatric neurology, Genetics

## Abstract

**Background:**

Pontocerebellar hypoplasia (PCH) describes a rare, heterogeneous group of neurodegenerative disorders mainly with a prenatal onset. Patients have severe hypoplasia or atrophy of cerebellum and pons, with variable involvement of supratentorial structures, motor and cognitive impairments. Based on distinct clinical features and genetic causes, current classification comprises 11 types of PCH.

**Main text:**

In this review we describe the clinical, neuroradiological and genetic characteristics of the different PCH subtypes, summarize the differential diagnosis and reflect on potential disease mechanisms in PCH. Seventeen PCH-related genes are now listed in the OMIM database, most of them have a function in RNA processing or translation. It is unknown why defects in these apparently ubiquitous processes result in a brain-specific phenotype.

**Conclusions:**

Many new PCH related genes and phenotypes have been described due to the appliance of next generation sequencing techniques. By including such a broad range of phenotypes, including non-degenerative and postnatal onset disorders, the current classification gives rise to confusion. Despite the discovery of new pathways involved in PCH, treatment is still symptomatic. However, correct diagnosis of PCH is important to provide suitable care and counseling regarding prognosis, and offer appropriate (prenatal) genetic testing to families.

## Background

Pontocerebellar Hypoplasia (PCH) was originally described as a heterogeneous group of prenatal onset neurodegenerative disorders, mainly but not exclusively affecting cerebellum and pons. The first report on PCH dates from 1917 [1]. Two reports that followed used the term ‘hypoplasia ponto- neocerebellaris’ to indicate the relative sparing of the cerebellar vermis compared to the cerebellar hemispheres [2, 3]. In 1928, the clinical characteristics of a patient with PCH were for the first time described by Krause [4]. He described a 16 months old, atrophic child with swallowing impairment, spasticity and jerky movements of the limbs; in retrospect this description and neuropathological profile fits PCH2. In 1961 the first patient with PCH and spinal motor neurodegeneration as seen in Werdnig-Hoffman disease (or spinal muscular atrophy, SMA) was reported [5]. More reports of similar patients followed, indicating that the combined occurrence of PCH and bulbo-spinal motor neuron degeneration was not an incidental finding but a distinct syndrome [6–8]. In 1990, Barth et al. described 7 affected children from 5 families from a Dutch genetic isolate, with PCH, microcephaly, severe extrapyramidal dyskinesia and absence of voluntary movement [9]. The extrapyramidal hyperkinesia in PCH was previously reported by Peiffer & Pfeiffer (1977) [10]. In 1993, a first classification of PCH was proposed that included two subtypes, PCH1 and PCH2 [11]. PCH1 is characterized by anterior horn degeneration in the spinal cord with muscle weakness and hypotonia. PCH2 is distinguished by neonatal jitteriness, incoordination of sucking and swallowing, lack of voluntary motor development and grasping and – in most patients- almost complete absence of cognitive development [11].

**Fig. 1:**
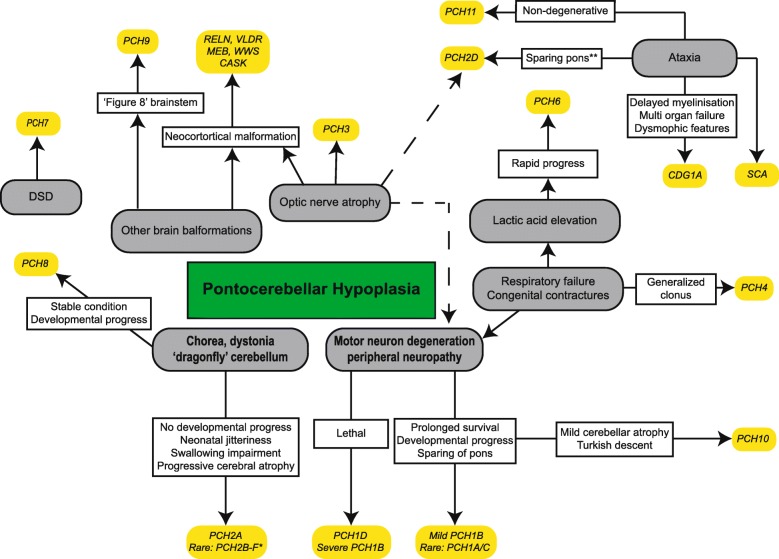
Simplified representation of main characteristic symptoms in PCH and similar conditions. DSD = Disorder of Sex Development, CC = Corpus callosum, MEB = Muscle-eye-brain disease, WWS = Walker Warburg syndrome, CDG1A = Congenital disorder of Glycosylation type Ia, SCA = Spinocerebellar ataxia. Note that exceptions are possible in most categories due to large phenotypic variability. *The strongest correlation of a dragonfly configuration of the cerebellum and dystonia is with PCH2A. In other PCH2 subtypes, that are all very rare, cerebellar hypoplasia may be less profound and extrapyramidal movement disorders absent. In PCH2E, brain MRI is initially normal; cerebellar atrophy becomes apparent in the first year of life. In addition, osteoporosis and scoliosis were reported in PCH2E.** (Relative) sparing of the pons can also be seen in milder affected patients with other PCH subtypes

Since the original description of PCH, the phenotype has been significantly broadened. Nine different subtypes were added to the classification of PCH, initially based upon distinct clinical, radiological or biochemical features (like optic atrophy, CSF lactate elevation), and later followed by the finding of associated gene defects (Table 1; Fig. 1). Inheritance of all PCH subtypes is autosomal recessive. Great clinical and neuroradiological variability exists within and between the different subtypes. Overall, PCH is a severe disorder with very limited or sometimes virtually absent developmental progress, and often early lethality. Most of the clinical neurological findings pertain to dysfunction of the cortex and basal ganglia, including severe intellectual disability, central motor impairments and epilepsy. Obvious cerebellar symptoms are very rarely reported in PCH, despite the severe cerebellar involvement shown by neuroimaging. The clinical features and genetics of the different subtypes, as well as the differential diagnosis of PCH and possible disease mechanisms, will be discussed separately below.

**Table 1:** Overview of PCH subtypes with associated gene defect. DSD = Disorders of Sex Development. * Imaging suggests postnatal onset of neurodegeneration in (a subset of) patients in this group

Subtype	Symptoms/ distinctive features in addition to PCH	Subcategory and gene (#OMIMnr)	Gene function	Key references
PCH1	Motor neuron degeneration, muscle weakness, hypotonia, respiratory insufficiency, congenital contractures	PCH1A: VRK1 (#607596)	Neuronal migration	[14-16]
		PCH1B: EXOSC3 (#614678)	mRNA degradation	[12, 13, 17]
		PCH1C: EXOSC8 (#616081)	mRNA degradation	[18]
		PCH1D: SLC25A46 (*610826)	Mitochondrial fission and fusion	[19, 20]
PCH2	Generalized clonus, impaired swallowing, Dystonia, chorea, progressive microcephaly	PCH2A: TSEN54 (#277470)	tRNA splicing	[21, 22, 24]
		PCH2B: TSEN2 (#612389)	tRNA splicing	[21, 25]
		PCH2C: TSEN34 (#612390)	tRNA splicing	[21]
		PCH2D^*^: SEPSECS (#613811)	Selenocysteine synthesis	[27-29]
		PCH2E: VPS53 (#615851)	Unknown	[30]
		PCH2F: TSEN15 (#617026)	tRNA splicing	[26]
PCH3	Facial dysmorphism, optic atrophy, cerebellar atrophy	PCLO^*^ (#608027)	Regulation synaptic protein & vesicle formation	[31-33]
PCH4	Severe form of PCH2 with congenital contractures and polyhydramnios	TSEN54 (#225753)	tRNA splicing	[21, 34]
PCH5	Severe form of PCH2 with congenital contractures and polyhydramnios (identical to PCH4)	TSEN54 (#610204)	tRNA splicing	[37, 38]
PCH6	Hypotonia, seizures, elevated CSF lactate, progressive supratentorial atrophy	RARS2^*^ (#611523)	Arginyl tRNA synthetase	[39, 40]
PCH7	DSD, thin corpus callosum, enlarged ventricles	TOE1 (#614969)	RNA processing	[52]
PCH8	Abnormal muscle tone, dystonia, ataxia, no/little disease progression. Non-degenerative.	CHMP1A (#614961)	Regulation INK4A	[54]
PCH9	Abnormal muscle tone, impaired swallowing, corpus callosum agenesis and ‘Fig. 8’ configuration of brainstem	AMPD2 (#615809)	Regulation GTP synthesis	[55-58]
PCH10	Abnormal muscle tone, seizures, motor neuron degeneration, mild cerebellar hypoplasia/atrophy	CLP1 (#615803)	tRNA splicing	[59-60]
PCH11	Non-progressive/ non-degenerative PCH.	TBC1D23 (# 617695)	Intracellular vesicle transport	[61, 62]

## Main text

### PCH1

**Fig. 2:**
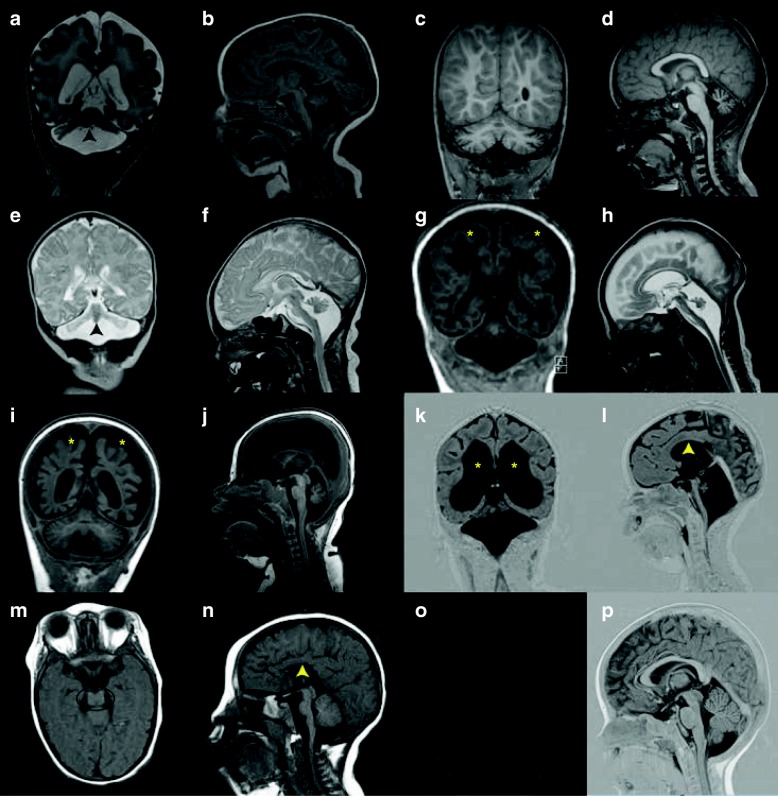
MR images in different PCH subtypes. **a-b**. Coronal (**a**) and midsagittal (**b**) image of severely affected PCH1B patient (age 3 weeks, homozygous for the p. G31A mutation in *EXOSC3*), showing severe hypoplasia of cerebellar hemisphere with relative sparing of the vermis (indicated by arrowhead). The ventral pons is reduced in size but not completely flattened (indicated by arrow). **c**-**d**. Coronal (**c**) and midsagittal (**d**) image of PCH1B patient (age 2,5 months) with milder *EXOSC3* mutations(the patient is homozygous for the p.D132A mutation). Cerebellar hypoplasia is very mild, with bilateral hyperintensities in the hili of the dentate nucleus. Size and shape of the ventral pons are normal. **e**-**f**. Coronal (**e**) and midsagittal (**f**) image of PCH2A patient (age 7 days, homozygous for the *TSEN54*, p.A307S mutation) with severe flattening of cerebellar hemispheres with relative sparing of the vermis (indicated by arrowhead) resulting in the ‘dragonfly’ configuration typical of PCH2A. This pattern is also seen in some PCH1 patients, as shown in 1A. The ventral pons is reduced in size but not completely flattened. **g**-**h**. Coronal (**g**) and midsagittal (**h**) image of PCH4 patient (age 6 weeks, compound heterozygous for the p. A307S mutation and a frameshift mutation in the *TSEN54* gene). Note severe flattening of pons and severe hypoplasia of the cerebellum without sparing of the vermis and profound supratentorial atrophy (indicated by asterisks). **i**-**j**. Coronal (**i**) and midsagittal (**j**) image of a PCH6 patient (age 6 months, compound heterozygous for mutations in the *RARS2* gene) with strikingly rapid progression of supratentorial atrophy (indicated by asterisks) and relatively slight atrophy of cerebellar hemispheres with normal pons and brainstem. **k**-**l**. Coronal (**k**) and midsagittal (**l**) image of a PCH7 patient (age 8 months, with biallelic mutations in the *TOE1* gene), showing severe pontocerebellar hypoplasia, equally affecting hemispheres and vermis. Note very thin corpus callosum and profound ventriculomegaly (indicated by asterisks and ^, respectively). **m**-**n**. Coronal and midsagittal image of a PCH9 patient (age 2 years, homozygous for a mutation in the *AMPD2* gene), showing characteristic ‘figure 8’ shaped brainstem and corpus callosum agenesis (indicated by a circle and arrowhead, respectively).**o**-**p**. Coronal and midsagittal image of a control

PCH1 is characterized by PCH with in addition bulbar and spinal motor neurodegeneration identical to spinal muscular atrophy (SMA) as seen in routinely stained spinal cord sections. Early reports describe PCH1 as a neonatally lethal disorder with polyhydramnios, congenital contractures, respiratory failure and severe muscle hypotonia [6, 11]. Later studies describe sparing of the ventral pons and survival into puberty, thereby broadening the clinical and neuroradiological spectrum of PCH1 [12, 13]. Currently, four genes are associated with PCH1 (PCH1A-D). Mutations in the vaccinia-related kinase 1 gene (*VRK1*, OMIM # 607596)) are a very rare cause of PCH1; *VRK1* mutations are described in one consanguineous PCH1 family of Ashkenazi Jewish origin [14]. The phenotype of these patients was atypical for PCH: patients had severe microcephaly from birth on while cognitive development seemed only mildly affected [14]. Later, VRK1 mutations turned out to be predominantly associated with motor neuron disease without PCH [15, 16]. Subsequent studies found pathogenic mutations in *EXOSC3* (OMIM # 614678) in roughly half of the patients with PCH1 [12, 13, 17]. *EXOSC3* encodes component 3 of the exosome complex, which is involved in mRNA degradation. Mutations in *EXOSC3* were clinically associated with prolonged survival (mean age at death was nine months in patients with mutations versus three months in patients without *EXOSC3* mutations) [13]. Respiratory failure, congenital contractures and polyhydramnios were rarely reported in the *EXOSC3* mutation group [13]. Cerebellar hypoplasia was variably present and the pons was unaffected in a part of the patient group, depending on the genotype (Fig. 2a-d) [12, 13]. Mutations in *EXOSC8* (OMIM # 61608), like *EXOSC3* encoding a component of the exosome complex, were found to cause psychomotor retardation, severe muscle weakness, spasticity, hearing and vision impairment and motor neuron degeneration in 22 patients from three families. Neuroimaging showed hypoplasia of the cerebellum and corpus callosum, cortical atrophy and immature myelinisation [18]. Mutations in *SLC25A46* (OMIM 610826) were shown to cause lethal pontocerebellar hypoplasia [19]. Mutations in this gene were also found to be the underlying genetic cause in the original Dutch PCH1 family, which was described by Barth to delineate the PCH1 phenotype as a distinct subtype of PCH [20]. With the identification of *SLC25A46* mutations, for the first time a genetic basis for the most severe spectrum of the PCH1 phenotype was revealed.

### PCH2

Probably PCH2A is the most prevalent and best characterized of all PCH subtypes. PCH2A is caused by homozygosity for a founder mutation (p.A307S) in the *TSEN54* gene (OMIM # 277470), which was identified by linkage analysis in 9 affected families from a Dutch genetic isolate [21]. Clinically, PCH2A is distinguished by generalized clonus and incoordination of sucking and swallowing in the neonate. The toddler and young child suffer from spasticity, dystonia/chorea and epilepsy and show a lack of voluntary motor development and grasping and almost complete absence of cognitive development [21–23]. Patients often suffer from feeding problems requiring PEG feeding. In PCH2A sleeping disorders, recurrent infections, apneas and problems in temperature regulation are reported in the majority of patients. Microcephaly, usually absent in the neonatal period, is progressive, and caused by supratentorial atrophy. Mean OFD at the age of 5 years was − 5.58 SD in a cohort of 33 patients studied by Sánchez and colleagues [24]. Life expectancy is reduced: more than 50% of patients die before puberty [24]. Homozygosity for the p.A307S mutation is strongly correlated with a ‘dragonfly’ configuration of the cerebellum on brain MRI, resulting from severely affected hemispheres (‘the wings’) and relative sparing of the vermis (‘ the body’; Fig. 2e-f) [22]. TSEN54 is part of the transfer-RNA (tRNA) splicing endonuclease complex: a complex involved in splicing of intron containing pre-tRNAs. The TSEN complex consists of four protein subunits: two catalytic (TSEN2 and TSEN34) and two structural (TSEN15 and TSEN54) ones. (Trotta 1997, Cell). Mutations in *TSEN2*, *TSEN34* and *TSEN15* rarely occur, resulting in a phenotype similar to PCH2 [22, 25, 26]. These subtypes are classified as PCH2B, PCH2C and PCH2F, respectively (respectively OMIM # 612389, # 612390 and # 617026) [26]. Classification of the PCH2 subtypes was done chronologically, based on the order of the identification of respective disease genes. PCH2D is caused by mutations in the *SEPSECS* gene (OMIM # 613811) and comprises a clinical spectrum with variable severity and some patients lack pontine hypoplasia [27–29]. In addition, patients with cognitive impairment and initial normal motor development with pediatric onset ataxia and motor decline have been described [29]. *VPS53* mutations (OMIM # 615851) were identified in four families of Jewish Moroccan ancestry and resulted in a PCH-like phenotype (PCH2E) [30]. VPS53 is one of the four subunits composing the Golgi-associated retrograde protein complex, involved in transporting endosomes to the trans-Golgi network. In all ten patients, pregnancy was uncomplicated and head circumference was normal at birth. During the first months of life, a delay in motor and cognitive development became apparent. Progressive spasticity, spastic quadriplegia, multiple contractures, progressive microcephaly and seizures evolved during infancy. Osteoporosis and scoliosis were reported. Initial brain imaging was normal, but cerebellar atrophy was noted in the first year of life followed by cerebral atrophy. All patients had profound mental retardation [30].

### PCH3

Pontocerebellar hypoplasia type 3, in the literature also referred to as cerebellar atrophy and progressive microcephaly (CLAM), is characterized by pontocerebellar hypoplasia/atrophy, thin corpus callosum, progressive microcephaly, seizures, small stature, facial dysmorphism and in some patients optic nerve atrophy. The extrapyramidal movement disorders that are typically seen in PCH2 are absent [31–33]. In 2015, it was shown that mutations in the *PCLO* (#608027) gene underlie PCH3 in a sibship form the Sultanate of Oman [31]. In 2009, a Turkish patient was described with a highly similar phenotype to the Omani patients [33]. The locus was mapped to the same region on chromosome 7q, in the region where the *PCLO* gene is located, but it remains unknown whether aberrations in this gene underlie the phenotype in this patient as well.

### PCH4&5

Pontocerebellar hypoplasia type 4, previously reported in the literature as olivopontocerebellar hypoplasia due to involvement of the inferior olivary nuclei, is at the severe end of *TSEN54*-related PCH spectrum. Clinically, PCH4 presents as a severe form of PCH2, with prenatal onset of symptoms including polyhydramnios and congenital contractures, prolonged neonatal clonus, hypertonia and primary hypoventilation requiring prolonged mechanical ventilation. Survival beyond the neonatal period is rare [22]. Neuropathology in PCH4 shows delayed maturation and reduced growth of the cerebral cortex. Pericerebral fluid accumulation is regularly seen, and probably results from shrinkage of the brain due to severe atrophy [22]. Extensive gliosis and a decreased number of neurons in the thalamus and caudate nucleus have been reported [34]. The cerebellar hemispheres were severely hypoplastic and devoid of Purkinje cells, while at most only remnants of the dentate nucleus could be identified [35, 36]. Hypoplasia of the cerebellar vermis is variable; but overall the vermis is more severely affected than in PCH2 (Fig. 2g-h) [22]. An important neuropathological difference with PCH2 is the lack of folding of the inferior olivary nuclei, resulting in a horseshoe shape [35]. The more severe clinical course in PCH4 is reflected by the type of mutation in *TSEN54.* In PCH2A, patients are homozygous for the p.A307S missense mutation. In PCH4, patients are compound heterozygous with on one allele this hypomorphic missense mutation and a premature stop- or splice site (loss-of-function) mutation on the other allele. The latter combination of mutations is more detrimental for protein function and results in a more severe phenotype of PCH4 compared to PCH2. The distinction between PCH4 and PCH5 was initially based upon the presence of prenatal seizures and a more severely affected vermis compared to the hemispheres in a single PCH5 family [37]. However, Namavar et al. showed that, as in PCH4, compound heterozygous *TSEN54* mutations, one being hypomorphic and the other being a loss-of-function mutation, underlie PCH5. The distinction of PCH5 as a separate subtype is therefore redundant [38].

### PCH6

The canonical PCH6 phenotype consists of severe early onset epilepsy, progressive global atrophy including pons and cerebellum, lactic acidosis and/or mitochondrial respiratory chain defects [39]. PCH6 is caused by mutations in the nuclear encoded mitochondrial Arginine tRNA-synthetase (*RARS2)*, which is responsible for catalyzing the specific attachment of Arginine to its cognate mitochondrial tRNA [40]. Currently, 31 patients with *RARS2* mutations (OMIM # 611523) have been reported [39–49]. None of them have biallelic null mutations, implicating that complete abolishment of mitochondrial tRNA-arg would be lethal. Splice site, nonsense or missense mutations are identified throughout the *RARS2* gene, while no clear genotype-phenotype correlation could be established [49]. All patients surviving the neonatal period had severe developmental delay, and almost all suffered from refractory epilepsy. The majority of patients had elevated lactic acid in plasma or CSF. Respiratory chain deficiency in fibroblasts or muscle could not be confirmed in all these patients. Brain MRI showed cerebellar hypoplasia/atrophy of variable severity. Some early MRIs were normal or showed mild vermal hypoplasia, with often rapidly progressive atrophy of supra- and infratentorial structures (Fig. 2i-j) [49].

### PCH7

PCH7 is characterized by the rare combination of PCH with disorders of sex development (DSD). Patients show a severe developmental delay, profound truncal hypotonia with hypertonic limbs and brisk deep tendon reflexes, and seizures. Disorders of sex development were most apparent in 46, XY patients, who showed undervirilisation ranging from almost completely differentiated female external genitalia to micropenis with hypoplastic scrotum. In some XY patients, uterine or ovarian remnants could be found, while others had atrophic testes or no gonadal tissue at all [50–53]. Clinical information on XX patients was provided for three affected individuals. Two had normal external female genitals, and one patient had a prominent clitoris. In one patient the ovaries could not be detected by ultrasound, while the uterus was present [52]. Patients of both sexes manifest profound hypoplasia of pons and cerebellum, a thin corpus callosum and enlarged ventricles with irregular borders as a consequence of reduced cerebral white matter (Fig. 2k-l) [51, 52]. Only one neuropathological report is available, provided by Anderson in 2011, which showed cerebral atrophy with a profound reduction in white matter. The cerebellar hemispheres were almost absent; only rudimentary folia devoid of neurons could be identified. The vermis was relatively spared. The ventral pons lacked pontine nuclei, while the pontine tegmentum was relatively normal. The inferior olivary nuclei could not be identified [51]. In 2017, biallelic mutations in *TOE1* (OMIM # 614969) were reported in 12 families with PCH7 [52].

### PCH8

Only three families with PCH8 have been reported. Patients have microcephaly, a severe developmental delay (although some patients were able to walk independently), dystonic posturing and/or choreiform movements. Some patients had (congenital) contractures and seizures. In contrast with the other PCH subtypes, PCH8 seems to be a stable neurologic condition without clear evidence of disease progression. Brain imaging showed profound pontocerebellar hypoplasia, a thin but fully formed corpus callosum and a decrease in cerebral white matter in all patients. In patients who had multiple brain MRIs, no clear signs of ongoing neurodegeneration were noted [54]. PCH8 might be considered as a ‘non-degenerative’ form of PCH and is caused by mutations in the *CHMP1A* gene (OMIM # 614961). CHMP1A is involved in chromatin modelling and cell proliferation [54].

### PCH9

PCH9 is characterized by progressive microcephaly, profound neurodevelopmental delay, cortical visual impairment, impaired swallowing, spasticity with neonatal clonus, brisk deep tendon reflexes and truncal hypotonia. Facial dysmorphisms are reported with dental abnormalities in a minority of patients. The presence of axonal neuropathy is reported in older patients and is probably age-dependent [55–57]. Brain MRI showed pontocerebellar hypoplasia, severe hypoplasia of the corpus callosum with, on axial images, a characteristic ‘figure 8’ shape of the mesencephalon, with hypoplastic cerebral peduncles (Fig. 2m-n) [55–58]. PCH9 is caused by mutations in the *AMPD2* gene (OMIM # 615809), which encodes one of the three adenosine monophosphate deaminases. Interestingly, mutations in the same gene have also been associated with spastic paraplegia 63 (SPG63; OMIM #615686).

### PCH10

Nine families of Turkish origin have been reported with PCH10 [59, 60].Mutations in *CLP1* has been identified as the causal gene defect (OMIM # 615803). CLP1 interacts with the TSEN complex and is also involved in tRNA splicing (see below). Clinical characteristics are a severe developmental delay, seizures, progressive spasticity, facial dysmorphism, microcephaly and signs of axonal neuropathy of both motor and sensory neurons. Brain MRI showed a thin corpus callosum, delay in myelinisation and hypoplasia of the ventral pons/thinning of the brainstem. Atrophy of the cerebellum is, if present at all, very mild compared to the other subtypes of PCH.

### PCH11

Seven families with PCH11 have been reported. Patients were homozygous for truncating or splice site mutations in the *TBC1D23* gene (OMIM # 617695) [61, 62]. PCH11 is characterized as another non-degenerative form of PCH and is characterized by a severe neurodevelopmental delay, microcephaly and hypotonia. Seizures were reported in 2/13 patients. Some patients were able to walk alone. Specific cerebellar symptoms like ataxia were reported in roughly half of the patients; this implies that some degree of voluntary movement was present in those patients. Brain MRI showed non-progressive hypoplasia of pons and cerebellum with hypoplasia of the corpus collosum [61, 62].

### Management and treatment

No curative treatment is available for any type of PCH. Treatment is symptomatic in all subtypes. PCH2A is the most prevalent subtype of PCH, and the only type in which adequate patient numbers allowed a systematic study of its clinical course. PCH2A patients often suffer from feeding problems starting immediately after birth requiring gavage or PEG feeding. Sleep apnoea, which is a frequently reported and life-threatening complication in PCH, and which may lead to sudden infant death, can be detected by sleep monitoring [24]. Seizures in PCH2A are difficult to treat, but a relatively good effect on phenobarbital and topiramate is reported (resulting in a reduction in seizure frequency in 11 out of 15 and 6 out of 7 patients, respectively) [24]. Choreathetoid movements are present in about 90% of patients. [22, 24] Opisthotonic posturing can be prevented by flexure of the large joints. An ergonomically shaped wheelchair and physiotherapy can be beneficial in achieving this. In one PCH2 patient, an improvement of dystonic crises and choreoathetosis upon treatment with Levodopa was reported [63]. Rhabdomyolysis and strongly elevated serum creatine kinase have been repeatedly reported and should be carefully monitored, especially during episodes of infection [23, 64–66]. Muscle biopsies and serum CK measurements of some PCH2 patients without any muscular symptoms showed evidence for a subclinical myopathy in a part of the PCH2 population [65].

PCH9 has been considered to be a potentially treatable disorder, because administration of a purine nucleotide precursor (AICAr) could rescue the phenotype at a cellular level. Follow-up experiments are needed, because it was shown that administering of AICAr tends to inhibit axonal growth in mouse cultures neurons [55].

### Differential diagnosis of pontocerebellar hypoplasia/atrophy

Cerebellar hypoplasia and cerebellar atrophy are both relatively common and nonspecific findings occurring in a very heterogeneous group of disorders [67]. Hypoplasia refers to a static condition in which the cerebellum has a normal shape but is too small, while atrophy refers to progressive loss of cerebellar tissue, resulting in progressive widening of interfolial spaces. The distinction between hypoplasia and atrophy may be easy to make on theoretical grounds; in practice however this can be challenging or even impracticable when based on a single brain imaging study.

Neuropathology reports in PCH2A mention both hypoplastic features with shortening of cerebellar folia with reduced branching (‘stunted folial growth’) and degenerative changes like thinning of the cerebellar cortex [35]. A mechanism of prenatal onset neurodegeneration is assumed, which is reflected by the ongoing postnatal neurodegeneration of the cerebral cortex, resulting in progressive microcephaly and widening of the lateral ventricles. The use of the term ‘hypoplasia’ in PCH is explained by historic reasons: at the time of the first descriptions of PCH, the concept of prenatal onset neurodegenerative disorders was yet unknown. Therefore, it was common to describe this congenital defect as ‘hypoplasia’.

Many metabolic disorders and genetic syndromes display cerebellar hypoplasia/atrophy. Careful assessment of the neuroimaging pattern is essential to evaluation. In PCH vermal growth is relatively spared compared to the cerebellar hemispheres, while in disorders like Joubert syndrome and Dandy-Walker malformation the vermis is mainly affected. The molar tooth sign, caused by elongation and a more horizontal course of the superior cerebellar peduncle, is characteristic of Joubert syndrome. While cerebellar hypoplasia has a broad differential diagnosis, the presence of pontine hypoplasia significantly narrows down the diagnostic options. Pontine hypoplasia affects the basis of the pons, and is mainly due to loss of ventral pontine neurons and transverse pontine fibers. However, the combination of pontine and cerebellar hypoplasia, does not automatically result in a diagnosis of PCH as discussed here. The use of the term ‘PCH’ is not uniform which may give rise to confusion. Some of the disorders that share pontine and cerebellar hypoplasia as a feature on MRI are discussed below (and Table 2).

**Table 2:** Differential diagnosis of Pontocerebellar Hypoplasia (PCH)

Disease	Distinctive features	Gene(s) involved	references
Congenital disorder of Glycosylation Ia (CDGIa)	Clinical: neonatal onset multi organ failure, dysmorphic features, ataxia. Microcephaly. MRI: global PCH with superimposed atrophy, supratentorial atrophy, ventriculomegaly, delayed myelinisation.	*PMM2*	[68, 69, 72]
*CASK*- related disorders	Clinical: facial dysmorphism, sensorineural hearing loss, ophthalmologic abnormalities. Developmental progress in a subgroup of patients. No MND or chorea. Progressive microcephaly. MRI: variable degree of PCH, equally affecting hemispheres and vermis. Cortical malformations can be present.	*CASK*	[73-76]
Tubulin defects	Clinical: DD, seizures. Progressive microcephaly. Optic atrophy in some cases. MRI: cortical malformations (eg lissencephaly, polymicrogyria) with cerebellar hypoplasia and brainstem malformations.	*TUBA1A*, *TUBB2B*, *TUBB3*, *TUBB5*, *TUBA8*	[77-79]
*RELN* & *VLDLR* mutations	Clinical: Severe DD, hypotonia, epilepsy, nystagmus. In *VLDLR*: non-progressive ataxia, quadrupedal gait. MRI: lissencephaly, severe PCH and abnormal hippocampus, milder in *VLDLR.*	*RELN, VLDLR*	[80-82]
α- dystroglycan related dystrophies (WWS, MEB, Fukuyama congenital muscular dystrophy)	Clinical: severe DD, muscle weakness with increased CK, ophthalmologic abnormalities. WWS at the severest end of the spectrum. MRI: wide spectrum of brain malformations including cobblestone lissencephaly, PCH, congenital hydrocephalus.	*POMT1, POMT2, POMGnT1, LARGE, FKTN, FKRP, ISPD, FKR, FKRP*	[83, 84, 85]
X-linked Hoyeraal-Hreidarsson syndrome	Clinical: IUGR, microcephaly, failure to thrive, progressive bone marrow failure, aplastic anemia, combined immunodeficiency, some symptoms of DC. MRI: PCH, delayed myelinisation, focal high intensities in brainstem and thalamus, subcortical calcifications.	*DKC1*	[86-87]
Pediatric onset Spinocerebellar Ataxia	Clinical: Ataxia, developmental progress, some cases with retinitis pigmentosa or cone-rod dystrophy. MRI: (ponto) cerebellar hypoplasia/atrophy, no supratentorial atrophy.	*ITPR1, ATXN7, ATXN2*	[88-92]
Extreme prematurity (< 32 weeks)	Clinical: motor and cognitive impairment of variable degree, autism spectrum disorders MRI: PCH, signs of cerebellar or cerebral injury, eg hemorrhage. Non progressive.	n/a	[95-97]

*MND* motor neuron degeneration, *DD* developmental delay, *CK* creatine kinase, *WWS* Walker-Warburg syndrome, *MEB* Musce Eye Brain disease, *IUGR* Intrauterine Growth Retardation, *DC* dyskeratosis congenita

#### Congenital disorder of glycosylation type 1A (CDG1A)

Congenital disorders of Glycosylation type 1a (CDG1a; OMIM #212065) present with global (ponto)cerebellar hypoplasia with superimposed atrophy on brain MRI [68, 67]. In addition to PCH, supratentorial atrophy with enlarged ventricles and delayed myelinisation can be present. [67, 68] Clinical presentation of CDG1a is highly variable: ranging from severe neonatal onset multi-organ failure to a stable neurological condition with moderate mental retardation and ataxia. Typical dysmorphic features are inverted nipples and abnormal fat distribution [69–71]. CDG1a is caused by autosomal recessive mutations in the *PMM2* gene and can be excluded by isoelectric focusing of transferrines. Cerebellar involvement is also reported in several other (rare) forms of CDG [72].

#### Pontocerebellar hypoplasia with neocortical malformations

##### CASK *– related disorders*

Loss of function mutations and (partial) deletions of the *CASK* gene, which is located on the X chromosome, are associated with microcephaly with pontine and cerebellar hypoplasia (MICPCH, OMIM # 300749) [73]. Pons and cerebellum can be severely hypoplastic, resembling PCH. Neocortical dysgenesis and malformations can be present in addition (e.g. reduction of number and complexity of frontal gyri and blurring of neocortical grey-white matter border) [73, 74]. Loss-of-function mutations and deletion often occur de novo. *CASK*- related PCH is clinically highly variable but is distinguished form PCH1 and PCH2 by the absence of motor neuron degeneration and chorea, respectively. *CASK* Patients show additional features like minor facial dysmorphisms (e.g. a round face with small chin, large nasal bridge), sensorineural hearing loss and ophthalmologic abnormalities [75]. The degree of cerebellar hypoplasia is variable, while the cerebellar hemispheres and vermis are equally involved without sparing of the vermis as seen in PCH1 and PCH2. The cerebellar hemispheres show asymmetry in some patients. Although developmental delay is severe, some patients achieve milestones like independent sitting or walking and speaking few single words [75].

##### Genes involved in the organization of microtubuli

Microtubuli play a key role in development of the brain by providing mechanical forces needed for cell division, axon guidance and neuronal migration [76, 77]. Mutations in several genes involved in tubulin formation are associated with a range of cortical malformations including lissencephaly and polymicrogyria, often combined with variable degrees of cerebellar hypoplasia and brain stem malformations [77]. *TUBA1A* mutations (OMIM # 611603) account for about 30% of patients with lissencephaly and cerebellar hypoplasia [78]. Other genes in the tubulin pathway that are associated with similar neuroradiological features are: *TUBB2B*, *TUBB3*, *TUBB5* and *TUBA8* [77].

##### RELN *and* VLDLR *mutations*

Autosomal recessive mutations in *RELN* (OMIM* 600514) result in lissencephaly with severe pontocerebellar hypoplasia and abnormal hippocampus [77, 79]. In contrast to PCH2, the cerebellar vermis is also very hypoplastic and often completely lacks foliation [78]. The Reelin signaling pathway is important for neuronal migration during development [77, 79]. The very low density lipid receptor (encoded by the *VLDLR* gene, OMIM# 224050) is an important player in this pathway and aberrations of *VLDLR* result in a similar, although generally milder, neurodevelopmental phenotype. Cortical malformations are milder, cerebellar ataxia is non-progressive and some patients have been described with a characteristic quadrupedal gait [80, 81].

##### α- dystroglycan related dystrophies

Defects in O-mannosyl-linked-glycosylation of α- dystroglycan underlie this group of heterogeneous congenital muscular dystrophies. The associated phenotypes form a wide clinical spectrum and are roughly divided in three main groups, depending on clinical severity: Fukuyama congenital muscular dystrophy, muscle-eye-brain disease and Walker-Warburg syndrome (WWS). Main characteristics are muscle weakness with increased serum CK, delayed motor development and severe cognitive impairment and opthalmological abnormalities (e.g. optic nerve atrophy, congenital glaucoma). Brain malformations include dysplastic supratentorial malformations (e.g. cobblestone lissencephaly or polymicrogyria) and cerebellar and pontine hypoplasia [82]. Also patients with a normal appearing brain MRI are reported [83]. Isolated cerebellar involvement occurs rarely [84].

#### X-linked Hoyeraal-Hreidarsson syndrome

X-linked Hoyeraal-Hreidarsson syndrome (XL-HHS) is characterized by intrauterine growth retardation, microcephaly, failure to thrive, developmental delay combined with progressive bone marrow failure, aplastic anemia and combined immunodeficiency [85, 86]. Neuroradiologic features are (ponto)cerebellar hypoplasia, hypoplastic corpus callosum, delayed myelination, focal high intensities in the brainstem and thalamus, and subcortical calcifications [87]. XL-HHS is caused by mutations in the *DKC1* gene (OMIM # 305000). *DKC1* is also involved in dyskeratosis congenital (DC), a syndrome characterized by bone marrow failure and a characteristic triad of abnormal skin pigmentation, oral leucoplakia and nail dystrophy [85, 86]. In XL-HHS, these characteristic mucocutaneous features of DC can be present [85, 87].

#### Spinocerebellar ataxias

Hypoplasia and/or atrophy of the cerebellum at early age can manifest in pediatric onset types of spinocerebellar ataxia (SCA). The SCA’s refer to a heterogeneous group of autosomal dominant inherited ataxia’s of varying severity and age of onset [88]. Brainstem involvement is not a regular finding in SCA, but has been reported in later disease stages of very severe infantile onset SCA2 and SCA7 [89–91]. Usually other findings like retinitis pigmentosa or cone-rod dystrophy are present in SCA2 and SCA7, that distinguish these conditions from PCH. More recently, it has been shown that mutations in *ITPR1*, can result in severe pontocerebellar hypoplasia, mimicking PCH [93]. Supratentorial atrophy was absent in this patient, while she clearly showed developmental progress.

#### *ATADA3* deletions/mutations

Biallelic deletions of the *ATAD3A* gene are associated with a lethal prenatal onset disorder characterized by polyhydramnios, contractures, severe pontocerebellar hypoplasia, microcephaly, abnormal basal ganglia and abnormalities of cortical gyration [94]. The *ATAD3* gene cluster comprises three genes that are arranged in tandem: *ATAD3C, ATAD3B* and *ATAD3A* [94]. Both homozygous deletions affecting the *ATAD3A/ ATAD3B* genes as well as some combinations of a null allele with missense mutations in *ATAD3A* are associated with this lethal phenotype. Milder mutations in *ATAD3A* and deletions involving *ATAD3B/ATAD3C* genes appear to be associated with a milder clinical course [94, 95]. The *ATAD3* gene cluster is involved in mtDNA organization and cholesterol metabolism [95].

#### Extreme prematurity

Extreme prematurity (gestational age < 32 weeks) is associated with an increased risk of cerebellar hypoplasia of various degrees. In severely affected patients, neuroradiologic imaging can mimic PCH [96]. The extremely rapid growth of the cerebellum in the third trimester results in an increased vulnerability of the premature cerebellum. Several (combined) mechanisms of preterm cerebellar injury are suggested, roughly consisting of ischemic of hemorrhagic destructive cerebellar lesions and cerebellar underdevelopment. In addition, effects of supratentorial injury on the development of cerebellum and brainstem trough involvement of cortico-cerebellar connections have been suggested [97, 98].

Brossard-Racine et al. reviewed the clinical outcome in preterm infants. Both motor and cognitive impairment are significantly associated with direct cerebellar injury. Also autistic features are often reported, especially when the cerebellar vermis is involved. The effects of indirect cerebellar injury (e.g. cerebellar underdevelopment associated with cerebral injury) on long-term development are less well studied and studies often show conflicting results [98].

### Pathomechanisms of PCH

#### PCH related to defects in tRNA splicing

**Fig. 3:**
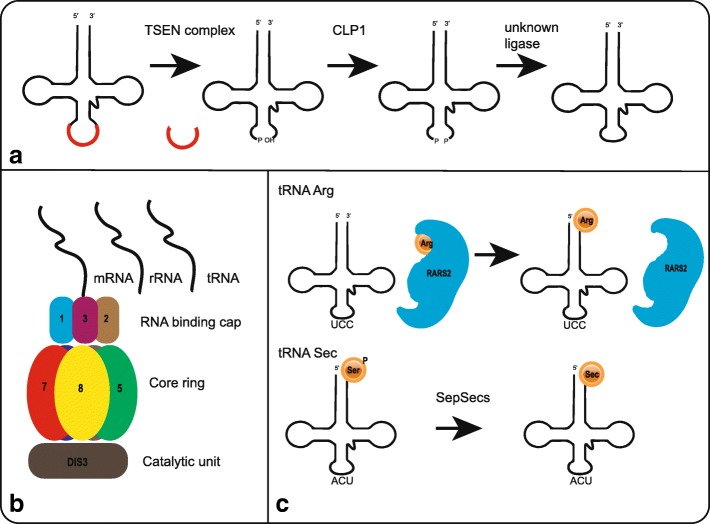
Schematic representation of main pathways involved in PCH. A. Schematic and simplified representation of tRNA splicing by the TSEN complex and CLP1. B. Schematic figure of the exosome complex including EXOSC3, located in the RNA binding cap, and EXOSC8, located in the core ring. The exosome complex processes mRNA, rRNA and presumably tRNA. C. Upper: charging of tRNA-Arg by RARS2. Lower: conversion of Sec-tRNA-Ser to Sec-tRNA-Sec by SEPSECS

Homozygosity for the p.A307S mutation in *TSEN54* is the most prevalent cause of PCH. As discussed above, TSEN54 is one of the four subunits of the transfer-RNA splicing endonuclease complex (TSEN-complex). Mutations in *TSEN2*, *TSEN34* and *TSEN15* occur very rarely. The TSEN complex is involved in the cleavage of intron containing tRNAs (Fig. 3a). tRNAs contain an anticodon sequence by which they recognize the codon of the mRNA template that is being translated. By transferring the required amino acid to the ribosomal complex for incorporation in the peptide chain, tRNAs are essential for protein translation. The human genome contains 506 genes that encode tRNAs; providing templates for multiple tRNAs for each codon. Only a minority of these tRNA genes (6%) contains an intron. For some amino acids however, the vast majority of tRNAs is intron containing (e.g. tRNA-Tyr (GTA) and tRNA-Ile (TAT)) (http://gtrnadb.ucsc.edu/). It is hypothesized that TSEN-complex defects result in impaired tRNA splicing and subsequently a decrease in protein translation. However, results of in vitro experiments studying the effect of the A307S mutation on tRNA splicing are ambiguous. Budde et al. reported no unspliced tRNA-Tyr products in patient fibroblasts, measured by RNA blot analysis [21].

CLP1*,* involved in PCH10, phosphorylates the 5′ ends of 3’ tRNA halves after splicing by the TSEN complex, thereby preparing re-ligation of the spliced tRNA halves by an unknown ligase. CLP1 is also known to interact with the TSEN complex; an interaction required for efficient tRNA splicing. It was shown that the R140H mutation in CLP1 hampers interaction with the TSEN complex, and thereby impairs tRNA splicing in vitro. In patient derived iNeurons, an accumulation of some pre-tRNAs with a corresponding reduction in mature tRNAs was found [59, 60].

#### PCH related to other defects in RNA processing and protein translation

Two subunits of the RNA exosome complex are involved in PCH. EXOSC3 functions as a RNA binding unit and is located in the cap of the complex, while EXOSC8 is one of the six subunits that composes the core ring. The exosome complex is important for degradation of mRNA and ribosomal RNA (rRNA) fragments and for maturation of certain rRNAs (Fig. 3b and see Fasken et al. for comprehensive summary) [99]. A role in tRNA degradation in human has been implied based on roles of the exosome complex in tRNA metabolism in yeast [100, 101]. Modeling of human *EXOSC3* mutations in *S. cerevisiae* affected pre-rRNA processing [102]. Why defects in rRNA processing specifically affects neurons remains unknown.

*SEPSECS*, mutated in PCH2D, encodes the O-phosphoseryl-tRNA(Sec) selenium transferase enzyme, which is needed for the final step in the synthesis of selenocystein. Selenocystein is essential for the synthesis of selenoproteins, but lacks its own tRNA-synthetase [27–29]. Therefore, to synthesize selenocystein, a Serine is transferred to a tRNA sec and is then via several steps converted to a Sec-tRNA-Sec complex (Fig. 3c) [103]. PCH6 is caused by mutations in the nuclear encoded mitochondrial Arginine tRNA-synthetase (*RARS2)*, which is responsible for catalyzing the specific attachment of Arginine to its cognate mitochondrial tRNA (Fig. 3c) [40]. In patient fibroblast, a reduction in the amount of mitochondrial tRNA-arg was found, but almost all molecules were charged. Authors suggest instability of the uncharged product to explain for this [40].

*TOE1*, mutated in PCH7, localizes to and is important for Cajal body maintenance, and a role of TOE1 in splicing of pre-mRNA was suggested [104]. Recent studies provide evidence for a role of TOE1 in the maturation of small nuclear RNAs and show accumulation of incompletely processed snRNAs in patient fibroblasts [52]. However, it remains unknown why defects in TOE1 specifically affect brain and gonadal development.

#### Other mechanisms in PCH

In some of the other PCH subtypes however, the link with RNA metabolism or protein translation is less clear. For example, *SLC25A46* (PCH1D) has a role in balancing mitochondrial fission and fusion and maintenance of mitochondrial cristae. Phenotypes associated with *SLC25A46*- mutations range from optic nerve atrophy and cerebellar atrophy to lethal PCH at the most severe end of the spectrum. Vaccinia related kinase1, encoded by *VRK1* and mutated in PCH1A, is implied in several fundamental cellular processes as coordination of cell division and regulation of Cajal body assembly [105, 106]. Knockdown of *VRK1* in a mouse model resulted in impaired neuronal migration in the cortex and influenced the neuronal progenitor cell cycle [107]. VPS53, involved in PCH2E, is one of the four subunits composing the Golgi-associated retrograde protein (GARP) complex. This complex is involved in retrograde transport to the trans-Golgi network [30]. *PCLO*, involved in PCH3, encodes piccolo, a presynaptic vesicle protein with a role in synaptic vesicle organization. In addition, a role in regulating presynaptic ubiquitination and proteostasis has been reported [108]. Piccolo was shown to be essential for the maintenance of synaptic integrity and loss of piccolo leads to degradation of multiple synaptic proteins and eventually to the disassembling of the synapse [109]. It was therefore suggested that the degeneration of synapses due to absence of piccolo might trigger an apoptotic signal in neurons in PCH3 patients [31]. CHMP1A (PCH8) has a function in chromatin modelling and cell proliferation. It is assumed that CHMP1A, via interaction with BMI1*,* has a role in inhibition of INK4, which is a negative regulator of stem cell proliferation [54]. PCH9 is caused by mutations in the *AMPD2* gene, which encodes one of the three adenosine monophosphate deaminases. AMPD2 converts AMP to IMP, which is then further processed to form guanine nucleotides. It was shown that defects in AMPD2 result in a depletion of guanine nucleotides and an accumulation of specific adenosine nucleotides [55]. This accumulation inhibited de novo purine synthesis by a negative feedback mechanism. Of the three AMPD paralogs, AMPD2 is predominantly expressed in the cerebellum, which possibly explains why cerebellar structures are most severely affected in PCH9 [55]. PCH11 is caused by truncating and splice site mutations in *TBC1D23* gene. TBC1D23 is involved in regulation of *trans-*Golgi membrane trafficking [61]. Intracellular vesicle transport was shown to be affected in patients fibroblasts and down regulation of *TBC1D23* in mice resulted in abnormal neural positioning in the cortex [62].

Alteration of RNA metabolism/ protein translation is a common mechanism behind at least some of the PCH subtypes. However, it remains unknown why the cerebellum is specifically affected by these defects in processes that seem vital to other organs as well. The third trimester is a period of tremendous growth for the developing cerebellum. It is hypothesized that the cerebellum is extremely vulnerable to alterations in RNA and/or protein metabolism in this specific time-window [23, 110]. This might partly explain for the cerebellum-specific involvement.

### Incidence of PCH

**Fig. 4:**
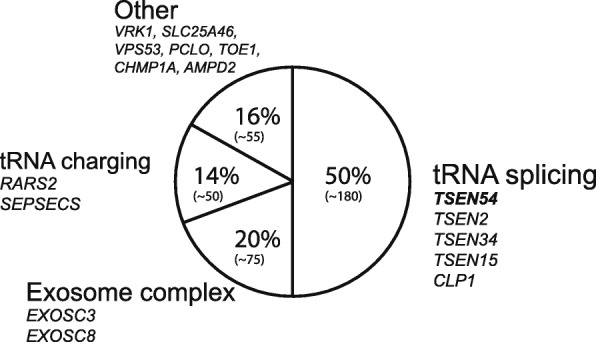
Pie-chart showing proportions of PCH patients per PCH pathway. Patient numbers are estimated based on published reports and reviews and depicted between brackets

PCH is a very rare disorder. The exact incidence of PCH is unknown. PCH2A caused by homozygosity for the p.A307S mutation in TSEN54 is the most frequent type of PCH (Fig. 4). The estimated incidence of PCH2A is < 1:200.000 [24]. We identified mutations in PCH related genes in 42 patients in routine diagnostic care between 2012 and 2017. Twenty out of those 42 patients were homozygous for the p.A307S mutation, 13 patients had mutations in *EXOSC3*, four patients harboured mutations in *RARS2,* one patient had *SEPSECS* mutations and one had mutations in *AMPD2*. We also identified hetero- or hemizygous *CASK* mutations in nine patients, indicating that this is a frequent cause of a condition similar to PCH.

### Genetic counselling and prenatal testing

Counselling regarding recurrence risk should be provided and reproductive options discussed. Preconception carrier screening is offered for PCH2A in a closed community in the Netherlands [111]. Since 2010, seven carrier couples were identified in this community. In families were the causal genetic defect is identified, preimplantation genetic diagnosis (PGD) or invasive prenatal testing should be offered for family planning. Prenatal detection of PCH by ultrasound is unreliable, since it often fails to detect cerebellar abnormalities at time of the routine 20 weeks screening for structural abnormalities [111–113].

## Discussion & Conclusion

Due to development and appliance of next generation sequencing techniques, many new PCH related genes and phenotypes have been described. However, there are still many patients with a clinical diagnosis of PCH, in whom no mutations in any of the known PCH related genes is identified.The current classification of PCH comprises a heterogeneous group of disorders, ranging from lethal conditions with prenatal onset as PCH1D and PCH4 to milder forms where disease progression is less devastating allowing survival into adolescence as (some subtypes of) PCH1B and PCH2D. Unfortunately, by including such a broad range of phenotypes, the current classification undermines two important aspects of the original description of PCH. Both nondegenerative conditions (e.g. PCH8 and PCH11) and disorders with a postnatal onset (e.g. PCH2D, PCH2E and PCH10) are included in the PCH classification, essentially ignoring the fact that PCH was originally defined as a disorder of prenatal onset neurodegeneration. PCH2D and PCH2E were initially described by the authors as progressive cerebellocerebral atrophy (PCCA and PCCA2, respectively), but eventually ended up as subtypes of PCH. PCH3 was originally referred to as cerebellar atrophy and progressive microcephaly (CLAM), because neuroimaging resembled cerebellar atrophy rather than hypoplasia. The patient numbers reported in PCH 3–11 are small (with only PCH6 due to *RARS2* mutations occurring relatively common), therefore the full clinical and radiological spectrum of these disorders is not completely revealed yet. The current classification of PCH is prone to lead to confusion, because at this point it is unclear which disorders should be classified as PCH and which should not.

Genotype-phenotype correlations are most clear in *TSEN54* (PCH2A and PCH4). Homozygosity for the p. A307S mutation in TSEN54 is strongly associated with a dragonfly configuration of the cerebellum, impaired swallowing and extrapyramidal movement disorders in infancy. In infants presenting with these symptoms and neuroradiological profile, prompt testing for this specific mutation is required. Patients compound heterozygous for the A307S mutation with a splice site or nonsense mutation on the other TSEN54 allele have a more severe phenotype characterized by congenital contractures and primary hypoventilation. In the majority of PCH patients, especially the milder ones and ones presenting with profound cerebral degeneration or other brain malformations, the diagnosis is less straightforward. Clinical, neuroradiological, metabolic and genetic data should be carefully evaluated and interpreted to get the correct diagnoses. It should not be overlooked that mutations in PCH related genes can also lead to milder phenotypes and might be revealed in adolescents or even young adults [114, 115]. When interpreting possible disease causing variants identified with whole exome sequencing, Genematcher can be a very useful tool to identify similar patients. Genematcher is an online tool where clinicians or researchers can enter their gene of interest and will be connected to others with an interest in the same gene [116]. Targeted gene panels have been developed to facilitate fast genetic diagnosis in PCH, but run the risk of being outdated soon because of the accelerated rate of new gene discovery.

The identification of novel genes resulted in new insights regarding the disease mechanism. Besides defects in RNA metabolism and protein synthesis, also other mechanisms like mitochondrial dysfunction (*SLC25A46*) and chromatin modelling (*CHMP1A*) are implied in PCH. Despite the discovery of new pathways involved in PCH, treatment is still symptomatic. PCH is a very rare disorder; in some subtypes only a few families are described. In addition, although clinical course is variable, the brain structure is already severely affect at birth in most children. These factors, together with the heterogeneous etiology in PCH, complicate the development of a targeted therapeutic strategy.
